# More Is Better: Selecting for Broad Host Range Bacteriophages

**DOI:** 10.3389/fmicb.2016.01352

**Published:** 2016-09-08

**Authors:** Alexa Ross, Samantha Ward, Paul Hyman

**Affiliations:** Department of Biology and Toxicology, Ashland University, AshlandOH, USA

**Keywords:** bacteriophage, host range, isolation protocol, phage therapy, bacteriophage ecology, bacteriophage evolution, phage–host interaction

## Abstract

Bacteriophages are viruses that infect bacteria. In this perspective, we discuss several aspects of a characteristic feature of bacteriophages, their host range. Each phage has its own particular host range, the range of bacteria that it can infect. While some phages can only infect one or a few bacterial strains, other phages can infect many species or even bacteria from different genera. Different methods for determining host range may give different results, reflecting the multiple mechanisms bacteria have to resist phage infection and reflecting the different steps of infection each method depends on. This makes defining host range difficult. Another difficulty in describing host range arises from the inconsistent use of the words “narrow” and especially “broad” when describing the breadth of the host range. Nearly all bacteriophages have been isolated using a single host strain of bacteria. While this procedure is fairly standard, it may more likely produce narrow rather than broad host range phage. Our results and those of others suggest that using multiple host strains during isolation can more reliably produce broader host range phages. This challenges the common belief that most bacteriophages have a narrow host range. We highlight the implications of this for several areas that are affected by host range including horizontal gene transfer and phage therapy.

## Introduction

Bacteriophages (phages) are viruses that infect bacteria. Phages are estimated to be the most abundant organisms on Earth with likely more than 10^31^ total individual phage on the planet (Chibani-Chennoufi et al., 2004; Abedon, 2008). The types (strains or species) of bacteria that a bacteriophage is able to infect is considered the host range of the phage in question (Hyman and Abedon, 2010). It is generally believed that most bacteriophages are only capable of infecting a narrow range of bacteria that are closely related (Ackermann and DuBow, 1987; Weinbauer, 2004). This is due to a combination of factors including specificity of phages’ host binding proteins, biochemical interactions during infection, presence of related prophages or particular plasmids (especially for phages adsorbing to pili) and bacterial phage-resistance mechanisms (Adams, 1959; Hyman and Abedon, 2010; Diaz-Munoz and Koskella, 2014).

Because many bacteriophages kill the bacterial cells they infect, phages provide a possible alternative to antibiotics. This use of bacteriophages, called phage therapy, was the first use proposed for phage soon after their discovery (Summers, 2012). While displaced by antibiotics in much of the world, the increasing frequency of antibiotic-resistant bacteria has led to renewed interest in phage therapy (Brussow, 2014; Kutter et al., 2015). Host range is a key property for phage therapy as well as the biology of bacteriophages in general. One of the advantages of phage therapy is that the specificity of phage–host range spares non-pathogenic microbes from being killed during treatment. Conversely, this same specificity limits the ability of a particular phage’s use to a small set of potential pathogens requiring more specific diagnosis (Nilsson, 2014; Mapes et al., 2016).

## Host Range Determination

Determining the host range of a specific phage can be somewhat difficult because measured host ranges depend on the technique used (Hyman and Abedon, 2010). Adams (1959, p. 440) states that “Host range is often, but not always, determined by success or failure of adsorption” but for phage therapy, host cell killing tends to be the key determination. Hyman and Abedon (2010) describe seven different host range types including adsorptive, penetrative (transductive), bactericidal, productive, plaquing, spotting, and lysogenic. Each host range type is dependent on phage successfully completing different steps of the infection process or determined by different methods of measuring host range. For example, a plaquing host range is found by determining whether a phage is able to form plaques on a particular species or strain of host bacteria. This is a common way to determine whether the phage can productively infect the bacteria. But not every host will allow plaquing even if the host does allow for a productive infection if infection only yields a limited number of progeny. Spot testing, in which a small volume of phage is placed on a growing lawn of bacteria, is perhaps the most common way of determining whether the phage is able to infect. While simple and rapid, this technique can sometimes cause false positives because of lysis of bacterial cells without phage infection. This is typically thought to occur when bacteria are lysed by a large number of phage adsorbing to the cell and lysing it (although it is unclear if this is a widespread mechanism) or by lysis due to residual endolysin or bacteriocins in the phage stock or other mechanisms (Abedon, 2011). Because of this, spotting host ranges tend to be an overestimate of what the true host range in terms of producing progeny is. See Khan and Nilsson (2015) for an experimental exploration of this topic. Unless explicitly indicated, for the remainder of this perspective we will use the term host range to refer to range of hosts that can produce progeny phage, the productive host range. For practical purposes, this is usually equivalent to the plaquing host range.

A second challenge to discussing host range is the use of the term “broad host range.” This term is used to describe a bacteriophage that can infect multiple species of bacteria (Greene and Goldberg, 1985; Uchiyama et al., 2008; Khan and Nilsson, 2015; Yu et al., 2016). But it is also used to describe a bacteriophage that can infect multiple strains of the same species of bacteria (Vinod et al., 2006; Gupta and Prasad, 2010; Anand et al., 2015; Xu et al., 2016). As well, the term polyvalent (or polyvalence) is sometime used equivalently to broad host range although polyvalent was previously more specifically reserved for “phages active on different bacterial genera” (Ackermann and DuBow, 1987). Whichever term is used, there is no standard as to how many or what percentage of strains/species tested must be infected for a phage to have a broad host range. For example, bacteriophage Mu is able to infect species of *Escherichia coli, Citrobacter freundii, Shigella sonnei, Enterobacter*, and *Erwinia* (Paolozzi and Ghelardini, 2006); staphylococcal phage ϕ812 infects 95% of 782 strains of *Staphylococcus aureus* and 43% of other *Staphylococcus* species tested (Pantucek et al., 1998); and bacteriophage P-27/HP infects 60% of 28 *S. aureus* isolates (Gupta and Prasad, 2010). All are described as having broad host range or being polyvalent. In addition to variation in numbers of bacteria species/strains tested, with a very few exceptions there are no standard collections of bacterial species. Instead, most researchers develop their own collections of bacteria usually including both previously described and newly isolated strains or species.

## Bacteriophage Isolation Protocols

Isolating phage can be a fairly simple process and for the most part researchers accomplish it in a very similar manner to that used by the earliest phage biologists (d’Hérelle and Smith, 1926; De Groat, 1927). The basic method is to obtain an environmental sample that is likely to contain or have been in contact with the targeted host bacteria. This can be raw fecal matter (Markel and Eklund, 1974; Jensen et al., 2015; Xu et al., 2016), sewage samples (Lin et al., 2010; Khan and Nilsson, 2015; Yu et al., 2016), water samples (Uchiyama et al., 2008; Lin et al., 2011), soil samples (Campos et al., 1978; Greene and Goldberg, 1985; Anand et al., 2015), samples taken from infected or healthy humans (Ackermann et al., 1975; Ronda et al., 1981), etc. Broth media or buffer is added to the samples and they are then filtered to remove the bacteria and other solid material. This filtrate is then added to a fresh culture of host bacteria and incubated overnight. In some cases, the sample is added directly to a growing bacterial culture without filtering. The next day, the culture is centrifuged to remove cell and other debris, and the supernatant is filtered to remove any remaining bacteria. This filtrate is then tested for the presence of phage by either spot or plaque testing. This procedure is so common as to be the basis for the standard method of screening for coliphage in water samples (International Organization for Standardization, 2000).

**FIGURE 1:**
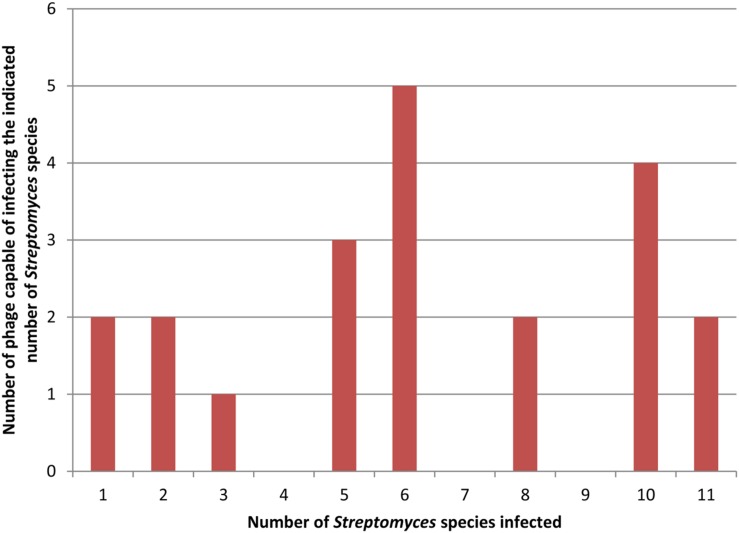
**Distribution of host range breadths of *Streptomyces* temperate phages.** Data for this figure is taken from Table 3 of Greene and Goldberg (1985). Twenty-one distinct phages were tested on 11 different host strains. In this figure both clear and turbid plaques (as indicated in the original results) are combined to indicate host susceptibility to a phage.

Most often, this procedure is performed using a single bacterial strain for phage isolation. The isolated phages are then tested against a collection of other bacterial strains and species to test host range breadth if host range is being measured. In many cases, this procedure produces phages with a narrow host range (Campos et al., 1978; Auad et al., 1997; Lu et al., 2003; Lin et al., 2011). The same procedure, however, can produce broader host range phages as well (Greene and Goldberg, 1985; Vinod et al., 2006; Uchiyama et al., 2008; Anand et al., 2015; Khan and Nilsson, 2015). When used to isolate many phages, both types of phages may be found together. For example, Greene and Goldberg (1985) screened about 700 soil samples for phages infecting *Streptomyces avermitilis* and found 57 phages that appeared unique by restriction analysis. They determined the host range of 21 of these that produced turbid plaques on *S. avermitilis*, suggesting a temperate life cycle. To do so, they used 10 additional *Streptomyces* species and found a diversity of host ranges based on plaque testing and as shown in **Figure 1**. Two phages were capable of infecting only a single host strain and two phages were capable of infecting all eleven host species with no obvious distribution of the phages infecting intermediate numbers of hosts. Jensen et al. (2015) found a similar mix of host range breadths by spot testing 12 novel phages against seven strains of *S. aureus*. **Figure 1** also shows the difficulty in determining the definitional borderline between narrow and broad host range as discussed above.

In order to isolate broader host range phage in a more controlled manner, we and other groups have modified the basic isolation procedure to use multiple hosts during phage isolation. Jensen et al. (1998) compared phages isolated on single hosts to phages isolated on pairs of hosts using *E. coli, Pseudomonas aeruginosa* and *Sphaerotilus natans* (all Gram-negative Proteobacteria). They found phages that could productively infect two or all three of the hosts in both the single- and double-host isolated phages. Mapes et al. (2016) developed a host range expansion (HRE) protocol to broaden the host range of *P. aeruginosa*-specific phages. They cultured a mixture of four phages with a mix of 16 different host strains and isolated individual phage strains by plaque isolation after multiple passages of phage mix onto fresh host mix. Over the course of 30 cycles host range was expanded as indicated by spot testing on both the 16 host strains and an additional 10 *P. aeruginosa* strains. We have isolated phages against *Enterococcus faecalis* using either one or a mixture of two host strains during isolation. As shown in **Table 1**, the two phages (AUEF4 and AUEF5) isolated using two strains had broader host ranges than the phage isolated using a single host (AUEF3) or another phage (VD13) previously isolated on the same host (Ackermann et al., 1975). The host ranges of AUEF4 and AUEF5 also included a strain of *E. faecium*.

**Table 1:** Host ranges of *Enterococcus faecalis* bacteriophages on *E. faecalis* and *E. faecium* strains.

		Isolated on 8413				Isolated on 8413 + OG1RF			
			
		VD13		AUEF3		AUEF4		AUEF5	
					
*E. faecalis* strain	Reference	Spot testing	Plaque	Spot testing	Plaque	Spot testing	Plaque	Spot testing	Plaque
8413 (ATCC 29200)	Ackermann et al., 1975	**+++**	**+**	**+++**	**+**	**+++**	**+**	**+++**	**+**
COM1	McBride et al., 2007	**-**	NT	**-**	NT	**-**	NT	**-**	NT
DS5	McBride et al., 2007	**+**	**-**	**-**	NT	**+**	**-**	**+**	**-**
DS16	McBride et al., 2007	**+**	**+^∗^**	**+++**	**+**	**+++**	**+**	**+++**	**+**
JH2	McBride et al., 2007	**-**	NT	**-**	NT	**-**	NT	**-**	NT
OG1RF	McBride et al., 2007	**+**	**-**	**+**	**-**	**-**	NT	**-**	NT
Pan7	McBride et al., 2007	**+**	**-**	**-**	NT	**+**	**-**	**+**	**-**
V583	McBride et al., 2007	**-**	NT	**-**	NT	**-**	NT	**-**	NT
EF-1	Yoong et al., 2004	**-**	NT	**-**	NT	**-**	NT	**-**	NT
EF-17	Yoong et al., 2004	**-**	NT	**+**	**-**	**+**	**-**	**+**	**-**
EF-24	Yoong et al., 2004	**-**	NT	**-**	NT	**-**	NT	**-**	NT
EF-25	Yoong et al., 2004	**-**	NT	**-**	NT	**+**	**+**	**+**	**+**
*E. faecium* strains								
EFSK2	Yoong et al., 2004	**-**	NT	**-**	NT	**-**	**+**	**+**	**+**
EFSK16	Yoong et al., 2004	**-**	NT	**-**	NT	**-**	NT	**-**	NT
EFSK33	Yoong et al., 2004	**-**	NT	**-**	NT	**-**	NT	**-**	NT


*^∗^Plaques seen but much smaller than plaques on 8413.*

*For spot testing ^+++^indicates complete lysis, ^+^indicates turbid lysis, ^-^indicates no lysis. For plaque testing ^+^indicates plaques seen, ^-^indicates no plaques observed. NT indicates phage/host combinations not tested as lack of any apparent lysis in spot testing suggests no interaction of that pair. For all phage, efficiency of plating was reduced on all hosts relative to 8413.*

Yu et al. (2016) have tested two sequential multiple host isolation methods after failing to find sufficiently broad host range phages using the simultaneous multi-host protocol described above. In method A, a mixed phage stock and host 1 is plated and plaques are allowed to form. All of these plaques are then collected, added to host 2, and then plated. This process can be continued with a third, fourth or more hosts and, in theory, phages from the last set of plaques collected should be able to infect all of the previous hosts. Similarly in method B, a phage stock is added to host 1 and the phages are allowed to infect and adsorb to the bacterial cells. The free phages and adsorbed phages are separated by centrifugation and the adsorbed phages are collected and allowed to grow on the first host for a few hours. The phages from this enrichment culture are then added to host 2. At least some of these phages will adsorb to and infect the bacterial cells. The free and adsorbed phages are again separated and the adsorbed phages are collected and allowed limited growth before transfer to another host. As with method A, this process can be continued and the last set of enriched phage should be able to infect all of the previous hosts. In both methods, single phage strains were then isolated using plaque purification. Yu et al. (2016) tested each method with several strains of *E. coli* and *P. aeruginosa* as well as one strain each of *P. putida* and *P. syringae*. Both methods produced phages able to infect all their *Pseudomonas* strains as well as some that could also infected *E. coli*.

## Implications and Conclusion

While there is some agreement as to the number of bacteria and viruses in various environments (Wommack and Colwell, 2000; Chibani-Chennoufi et al., 2004; Suttle, 2007), one of the unresolved questions of phage ecology is how frequently a particular bacteriophage encounters a susceptible host outside of the laboratory (Koskella and Meaden, 2013). The relative ease of finding phages capable of infecting multiple strains or species of bacteria, as in the studies cited here, suggests that this may be less of an issue as many phages may be capable of infecting multiple types of hosts. It may also be an error to consider host range as a stable property with a particular host always in or out of a phage’s host range (Hyman and Abedon, 2010; Koskella and Meaden, 2013) as hosts may evolve phage resistance and phages can evolve to overcome this resistance (Stern and Sorek, 2011; Samson et al., 2013; Chaturongakul and Ounjai, 2014). This has many implications for phage biology as well as for practical applications of phages.

The unresolved question of how often a phage finds a susceptible host has implications for the role of host range in horizontal gene transfer (HGT) of the bacterial hosts. Bacteriophages can move their own genome between host genomes by forming lysogens in the hosts. Lysogenic conversion, the expression of phage genes from the prophage, plays a role in pathogenesis of several bacterial species (Brüssow et al., 2004; Hyman and Abedon, 2008; Fortier and Sekulovic, 2013). For example, *E. coli* strains such as *E. coli* O157:H7 that produce Shiga-toxin are lysogens for a lambdoid prophage that contains the genes for the toxin (Unkmeir and Schmidt, 2000; Hyman and Abedon, 2008). Likewise, transduction, the movement of bacterial genome fragments via a phage capsid, is an important mechanism of HGT (Brüssow, 2008; Hendrix and Casjens, 2008; Kelly et al., 2009). Transductive host range describes which bacteria are susceptible to HGT by a particular phage and differs from productive host range in that the phage need not be able to replicate on the host but only needs to insert DNA into the recipient cell (Hyman and Abedon, 2010). As phages may utilize HGT themselves to evolve by exchanging segments between infecting phage and prophages, host range in general may similarly affect phage evolution (Campbell, 1988; Labrie and Moineau, 2007).

Broad host range phages are seen as more useful as well in some applications of bacteriophages. For phage therapy, a broad host range phage that kills multiple species of bacteria would be the equivalent of a broad spectrum antibiotic. Currently, multiple phage species are often mixed into a cocktail in order to treat several different bacteria that may be the cause of infections (Gill and Hyman, 2010; Kutateladze and Adamia, 2010; Lu and Koeris, 2011; Chan and Abedon, 2012). A smaller number of broad host range phages could be more useful than a larger number of narrow host range phages. On the other hand, the findings of studies such as that shown in **Figure 1** may mean that even phages isolated on a single host may unexpectedly infect other hosts. This might partly obviate one of the commonly cited advantages of phage therapy, host specificity that spares normal flora bacteria (Loc-Carrillo and Abedon, 2011; Nilsson, 2014). Other potential uses of phages that depend on host specificity such as using phage to deliver therapeutic or other genes to bacteria (Westwater et al., 2003; Clark et al., 2012) and the use of bacteriophage and bacteriophage proteins as biosensors for pathogenic bacteria (Zourob and Ripp, 2010; Singh et al., 2013; Peltomaa et al., 2016) might be similarly affected.

In conclusion, bacteriophage host range is not a fixed property of each species of bacteriophage. Rather, it is one that can evolve over time and can show unexpected plasticity. Modifying procedures and growth conditions can favor the isolation of novel phages with broader host ranges. Narrow host range cannot be assumed but must be tested just as life cycle, transduction potential and carriage of toxin genes are screened for when phage are isolated for other applications. We strongly encourage the use of plaque testing for determining host range when testing for phage therapy and related applications rather than spot testing as plaque formation shows that phages are capable of productive infection. Unless one is certain of being able to use a sufficient dose of phage to attack all bacteria in a single treatment, phage progeny production is needed to kill a bacterial population. Furthermore, for uses such as phage therapy, testing on clinical isolates is better than testing on laboratory host strains. Lastly, we encourage more judicious use of the term broad host range and recommend reserving its use for phages that have been shown to infect multiple bacterial species at a minimum and preferably, multiple genera.

## Author Contributions

All three authors substantially contributed to this work and all have read and approved it. SW isolated AUEF 4 and AUEF5, determined their host ranges and completed the primary analysis of all four phages. AR measured host ranges, did the literature review and primary preparation of the manuscript. PH conceived of and designed the host range project as well as helping draft and editing the manuscript.

## Conflict of Interest Statement

The authors declare that the research was conducted in the absence of any commercial or financial relationships that could be construed as a potential conflict of interest.

## References

[B1] Abedon S. T. (2008). “Phages, ecology, evolution,” in *Bacteriophage Ecology*, ed. Abedon S. T. (Cambridge: Cambridge University Press), 1–28.

[B2] Abedon S. T. (2011). Lysis from without. *Bacteriophage* 1 46–49. 10.4161/bact.1.1.13980 21687534 PMC3109453

[B3] Ackermann H. W. Caprioli T. Kasatiya S. S. (1975). A large new *Streptococcus* bacteriophage. *Can. J. Microbiol.* 21 571–574. 10.1139/m75-080 1122429

[B4] Ackermann H.-W. DuBow M. S. (1987). “Phage multiplication,” in *Viruses of Prokaryotes: General Properties of Bacteriophages* Vol. 1 eds Ackermann H.-W. DuBow M. S. (Boca Raton, Florida: CRC Press, Inc.), 49–85.

[B5] Adams M. H. (1959). *Bacteriophages.* New York, NY: InterScience.

[B6] Anand T. Vaid R. K. Bera B. C. Barua S. Riyesh T. Virmani N. (2015). Isolation and characterization of a bacteriophage with broad host range, displaying potential in preventing bovine diarrhoea. *Virus Genes* 51 315–321. 10.1007/s11262-015-1222-9 26174698

[B7] Auad L. de Ruiz holgado A. A. P. Forsman P. Alatossava T. Raya R. R. (1997). Isolation and characterization of a new *Lactobacillus delbrueckii* ssp.bulgaricus temperate bacteriophage. *J. Dairy Sci.* 80 2706–2712. 10.3168/jds.S0022-0302(97)76231-3

[B8] Brüssow H. (2008). “Phage-bacterium co-evolution and its implication for bacterial pathogenesis,” in *Horizontal Gene Transfer in the Evolution of Pathogenesis*, eds Hensel M. Schmidt H. (Cambridge: Cambridge University Press), 49–77.

[B9] Brussow H. (2014). Phage therapy: quo vadis? *Clin. Infect. Dis.* 58 535–536. 10.1093/cid/cit776 24270168

[B10] Brüssow H. Canchaya C. Hardt W. D. (2004). Phages and the evolution of bacterial pathogens: from genomic rearrangements to lysogenic conversion. *Microbiol. Mol. Biol. Rev.* 68 560–602. 10.1128/MMBR.68.3.560-602.2004 15353570 PMC515249

[B11] Campbell A. (1988). “Phage evolution and speciation,” in *The Bacteriophages* Vol. 1 ed. Calendar R. (New York, NY: Plenum Press), 1–14.

[B12] Campos J. M. Geisselsoder J. Zusman D. R. (1978). Isolation of bacteriophage MX4, a generalized transducing phage for *Myxococcus xanthus*. *J. Mol. Biol.* 119 167–178. 10.1016/0022-2836(78)90431-X 416222

[B13] Chan B. K. Abedon S. T. (2012). Phage therapy pharmacology: phage cocktails. *Adv. Appl. Microbiol.* 78 1–23. 10.1016/B978-0-12-394805-2.00001-4 22305091

[B14] Chaturongakul S. Ounjai P. (2014). Phage-host interplay: examples from tailed phages and Gram-negative bacterial pathogens. *Front. Microbiol.* 5:442 10.3389/fmicb.2014.00442 PMC4138488 25191318

[B15] Chibani-Chennoufi S. Bruttin A. Dillmann M. L. Brüssow H. (2004). Phage-host interaction: an ecological perspective. *J. Bacteriol.* 186 3677–3686. 10.1128/JB.186.12.3677-3686.2004 15175280 PMC419959

[B16] Clark J. Abedon S. T. Hyman P. (2012). “Phages as therapeutic delivery vehicles,” in *Bacteriophages in Health and Disease*, eds Hyman P. Abedon S. T. (Wallingford: CABI Press), 86–100.

[B17] De Groat A. F. (1927). The bacteriophage: a method of isolation. *J. Immunol.* 14 175–179.

[B18] d’Hérelle F. Smith G. H. (1926). *The Bacteriophage and Its Behavior [English translation].* Baltimore: The Williams &Wilkins Co.

[B19] Diaz-Munoz S. L. Koskella B. (2014). Bacteria-phage interactions in natural environments. *Adv. Appl. Microbiol.* 89 135–183. 10.1016/B978-0-12-800259-9.00004-4 25131402

[B20] Fortier L. C. Sekulovic O. (2013). Importance of prophages to evolution and virulence of bacterial pathogens. *Virulence* 4 354–365. 10.4161/viru.24498 23611873 PMC3714127

[B21] Gill J. J. Hyman P. (2010). Phage choice, isolation, and preparation for phage therapy. *Curr. Pharm. Biotechnol.* 11 2–14. 10.2174/138920110790725311 20214604

[B22] Greene J. Goldberg R. B. (1985). Isolation and preliminary characterization of lytic and lysogenic phages with wide host range within the streptomycetes. *J. Gen. Microbiol.* 131 2459–2465. 10.1099/00221287-131-9-2459 2999304

[B23] Gupta R. Prasad Y. (2010). Efficacy of polyvalent bacteriophage P-27/HP to control multidrug resistant *Staphylococcus aureus* associated with human infections. *Curr. Microbiol.* 62 255–260. 10.1007/s00284-010-9699-x 20607539

[B24] Hendrix R. W. Casjens S. R. (2008). “The role of bacteriophages in the generation and spread of bacterial pathogens,” in *Horizontal Gene Transfer in the Evolution of Pathogenesis*, eds Hensel M. Schmidt H. (Cambridge: Cambridge University Press), 79–112.

[B25] Hyman P. Abedon S. T. (2008). “Phage ecology of bacterial pathogenesis,” in *Bacteriophage Ecology*, ed. Abedon S. T. (Cambridge: Cambridge University Press), 353–385.

[B26] Hyman P. Abedon S. T. (2010). Bacteriophage host range and bacterial resistance. *Adv. Appl. Microbiol.* 70 217–248. 10.1016/S0065-2164(10)70007-1 20359459

[B27] International Organization for Standardization (2000). *Water Quality – Detection and Enumeration of Bacteriophages, Part 2: Enumeration of Somatic Coliphages.* *ISO* 10705-2:2000(E) Geneva: International Organization for Standardization.

[B28] Jensen E. C. Schrader H. S. Rieland B. Thompson T. L. Lee K. W. Nickerson K. W. (1998). Prevalence of broad-host-range lytic bacteriophages of *Sphaerotilus* natans, *Escherichia coli*, and *Pseudomonas aeruginosa*. *Appl. Environ. Microbiol.* 64 575–580. 9464396 10.1128/aem.64.2.575-580.1998 PMC106085

[B29] Jensen K. C. Hair B. B. Wienclaw T. M. Murdock M. H. Hatch J. B. Trent A. T. (2015). Isolation and host range of bacteriophage with lytic activity against methicillin-resistant *Staphylococcus aureus* and potential use as a fomite decontaminant. *PLoS ONE* 10:e0131714 10.1371/journal.pone.0131714 PMC4488860 26131892

[B30] Kelly B. G. Vespermann A. Bolton D. J. (2009). The role of horizontal gene transfer in the evolution of selected foodborne bacterial pathogens. *Food Chem. Toxicol.* 47 951–968. 10.1016/j.fct.2008.02.006 18420329

[B31] Khan M. M. Nilsson A. S. (2015). Isolation of phages for phage therapy: a comparison of spot tests and efficiency of plating analyses for determination of host range and efficacy. *PLoS ONE* 10:e0118557 10.1371/journal.pone.0118557 PMC4356574 25761060

[B32] Koskella B. Meaden S. (2013). Understanding bacteriophage specificity in natural microbial communities. *Viruses* 5 806–823. 10.3390/v5030806 23478639 PMC3705297

[B33] Kutateladze M. Adamia R. (2010). Bacteriophages as potential new therapeutics to replace or supplement antibiotics. *Trends Biotechnol.* 28 591–595. 10.1016/j.tibtech.2010.08.001 20810181

[B34] Kutter E. M. Kuhl S. J. Abedon S. T. (2015). Re-establishing a place for phage therapy in western medicine. *Future Microbiol.* 10 685–688. 10.2217/fmb.15.28 26000644

[B35] Labrie S. J. Moineau S. (2007). Abortive infection mechanisms and prophage sequences significantly influence the genetic makeup of emerging lytic lactococcal phages. *J. Bacteriol.* 189 1482–1487. 10.1128/JB.00728-07 17041060 PMC1797345

[B36] Lin L. Han J. Ji X. Hong W. Huang L. Wei Y. (2011). Isolation and characterization of a new bacteriophage MMP17 from *Meiothermus*. *Extremophiles* 15 253–258. 10.1007/s00792-010-0354-z 21225300

[B37] Lin N. T. Chiou P. Y. Chang K. C. Chen L. K. Lai M. J. (2010). Isolation and characterization of ϕAB2: a novel bacteriophage of *Acinetobacter baumannii*. *Res. Microbiol.* 161 308–314. 10.1016/j.resmic.2010.03.007 20385229

[B38] Loc-Carrillo C. Abedon S. T. (2011). Pros and cons of phage therapy. *Bacteriophage* 1 111–114. 10.4161/bact.1.2.14590 22334867 PMC3278648

[B39] Lu T. K. Koeris M. S. (2011). The next generation of bacteriophage therapy. *Curr. Opin. Mirobiol.* 14 524–531. 10.1016/j.mib.2011.07.028 21868281

[B40] Lu Z. Breidt F. J. Fleming H. P. Altermann E. Klaenhammer T. R. (2003). Isolation and characterization of a *Lactobacillus plantarum* bacteriophage, ϕJL-1, from a cucumber fermentation. *Int. J. Food Microbiol.* 84 225–235. 10.1016/S0168-1605(03)00111-9 12781945

[B41] Mapes A. C. Trautner B. W. Liao K. S. Ramig R. F. (2016). Development of expanded host range phage active on biofilms of multi-drug resistant *Pseudomonas aeruginosa*. *Bacteriophage* 6:e1096995 10.1080/21597081.2015.1096995 PMC4836484 27144083

[B42] Markel D. E. Eklund C. (1974). Isolation, characterization, and classification of three bacteriophage isolates for the genus *Levinea*. *Int. J. Syst. Bacteriol.* 24 230–234. 10.1099/00207713-24-2-230

[B43] McBride S. M. Fischetti V. A. Leblanc D. J. Moellering R. C. Jr. Gilmore M. S. (2007). Genetic diversity among *Enterococcus faecalis*. *PLoS ONE* 2:e582 10.1371/journal.pone.0000582 PMC1899230 17611618

[B44] Nilsson A. S. (2014). Phage therapy–constraints and possibilities. *Ups. J. Med. Sci.* 119 192–198. 10.3109/03009734.2014.902878 24678769 PMC4034558

[B45] Pantucek R. Rosypalova A. Doskar J. Kailerova J. Ruzicková V. Borecka P. (1998). The polyvalent staphylococcal phage ϕ812: its host-range mutants and related phages. *Virology* 246 241–252. 10.1006/viro.1998.9203 9657943

[B46] Paolozzi L. Ghelardini P. (2006). “The bacteriophage Mu,” in *The Bacteriophages*, eds Calendar R. Abedon S. T. (Oxford: Oxford University Press), 469–496.

[B47] Peltomaa R. Lopez-Perolio I. Benito-Pena E. Barderas R. Moreno-Bondi M. C. (2016). Application of bacteriophages in sensor development. *Anal. Bioanal. Chem.* 408 1805–1828. 10.1007/s00216-015-9087-2 26472318

[B48] Ronda C. Lopez R. Garcia E. (1981). Isolation and characterization of a new bacteriophage, Cp-1, infecting *Streptococcus pneumoniae*. *J. Virol.* 40 551–559. 6275103 10.1128/jvi.40.2.551-559.1981 PMC256658

[B49] Samson J. E. Magadan A. H. Sabri M. Moineau S. (2013). Revenge of the phages: defeating bacterial defences. *Nat. Rev. Microbiol.* 11 675–687. 10.1038/nrmicro3096 23979432

[B50] Singh A. Poshtiban S. Evoy S. (2013). Recent advances in bacteriophage based biosensors for food-borne pathogen detection. *Sensors (Basel)* 13 1763–1786. 10.3390/s130201763 23364199 PMC3649382

[B51] Stern A. Sorek R. (2011). The phage-host arms race: shaping the evolution of microbes. *Bioessays* 33 43–51. 10.1002/bies.201000071 20979102 PMC3274958

[B52] Summers W. C. (2012). The strange history of phage therapy. *Bacteriophage* 2 130–133. 10.4161/bact.20757 23050223 PMC3442826

[B53] Suttle C. A. (2007). Marine viruses – major players in the global ecosystem. *Nat. Rev. Microbiol.* 5 801–812. 10.1038/nrmicro1750 17853907

[B54] Uchiyama J. Rashel M. Maeda Y. Takemura I. Sugihara S. Akechi K. (2008). Isolation and characterization of a novel *Enterococcus faecalis* bacteriophage ϕEF24C as a therapeutic candidate. *FEMS Microbiol. Lett.* 278 200–206. 10.1111/j.1574-6968.2007.00996.x 18096017

[B55] Unkmeir A. Schmidt H. (2000). Structural analysis of phage-borne stx genes and their flanking sequences in shiga toxin-producing *Escherichia coli* and *Shigella dysenteriae* type 1 strains. *Infect. Immun.* 68 4856–4864. 10.1128/IAI.68.9.4856-4864.2000 10948097 PMC101682

[B56] Vinod M. G. Shivu M. M. Umesha K. R. Rajeeva B. C. Krohne G. Karunasagar I. (2006). Isolation of *Vibrio harveyi* bacteriophage with a potential for biocontrol of luminous vibriosis in hatchery environments. *Aquaculture* 255 117–124. 10.1016/j.aquaculture.2005.12.003

[B57] Weinbauer M. G. (2004). Ecology of prokaryotic viruses. *FEMS Microbiol. Rev.* 28 127–181. 10.1016/j.femsre.2003.08.001 15109783

[B58] Westwater C. Kasman L. M. Schofield D. A. Werner P. A. Dolan J. W. Schmidt M. G. (2003). Use of genetically engineered phage to deliver antimicrobial agents to bacteria: an alternative therapy for treatment of bacterial infections. *Antimicrob. Agents Chemother.* 47 1301–1307. 10.1128/AAC.47.4.1301-1307.2003 12654662 PMC152521

[B59] Wommack K. E. Colwell R. R. (2000). Virioplankton: viruses in aquatic ecosystems. *Microbiol. Mol. Biol. Rev.* 64 69–114. 10.1128/MMBR.64.1.69-114.2000 10704475 PMC98987

[B60] Xu J. Chen M. He L. Zhang S. Ding T. Yao H. (2016). Isolation and characterization of a T4-like phage with a relatively wide host range within *Escherichia coli*. *J. Basic Microbiol.* 56 405–421. 10.1002/jobm.201500440 26697952

[B61] Yoong P. Schuch R. Nelson D. Fischetti V. A. (2004). Identification of a broadly active phage lytic enzyme with lethal activity against antibiotic-resistant *Enterococcus faecalis* and *Enterococcus faecium*. *J. Bacteriol.* 186 4808–4812. 10.1128/JB.186.14.4808-4812.2004 15231813 PMC438584

[B62] Yu P. Mathieu J. Li M. Dai Z. Alvarez P. J. (2016). Isolation of polyvalent bacteriophages by sequential multiple-host approaches. *Appl. Environ. Microbiol.* 82 808–815. 10.1128/AEM.02382-15 26590277 PMC4725286

[B63] Zourob M. Ripp S. (2010). “Bacteriophage-based biosensors,” in *Recognition Receptors in Biosensors*, ed. Zourob M. (Berlin: Springer Science), 415–448.

